# Pansharpening with a Guided Filter Based on Three-Layer Decomposition

**DOI:** 10.3390/s16071068

**Published:** 2016-07-12

**Authors:** Xiangchao Meng, Jie Li, Huanfeng Shen, Liangpei Zhang, Hongyan Zhang

**Affiliations:** 1 School of Resource and Environmental Sciences, Wuhan University, Luoyu Road, Wuhan 430079, China; mengxc@whu.edu.cn; 2 State Key Laboratory of Information Engineering in Surveying, Mapping and Remote Sensing, Wuhan University, Luoyu Road, Wuhan 430079, China; aaronleecool@hotmail.com (J.L.); zlp62@whu.edu.cn (L.Z.); zhanghongyan@whu.edu.cn (H.Z.); 3 Beijing Advanced Innovation Center for Imaging Technology, Capital Normal University, Beijing 100048, China; 4 Collaborative Innovation Center of Geospatial Technology, Luoyu Road, Wuhan 430079, China

**Keywords:** pansharpening, guided filter, three-layer decomposition, panchromatic (PAN), multispectral (MS)

## Abstract

State-of-the-art pansharpening methods generally inject the spatial structures of a high spatial resolution (HR) panchromatic (PAN) image into the corresponding low spatial resolution (LR) multispectral (MS) image by an injection model. In this paper, a novel pansharpening method with an edge-preserving guided filter based on three-layer decomposition is proposed. In the proposed method, the PAN image is decomposed into three layers: A strong edge layer, a detail layer, and a low-frequency layer. The edge layer and detail layer are then injected into the MS image by a proportional injection model. In addition, two new quantitative evaluation indices, including the modified correlation coefficient (MCC) and the modified universal image quality index (MUIQI) are developed. The proposed method was tested and verified by IKONOS, QuickBird, and Gaofen (GF)-1 satellite images, and it was compared with several of state-of-the-art pansharpening methods from both qualitative and quantitative aspects. The experimental results confirm the superiority of the proposed method.

## 1. Introduction

With the rapid development of satellite sensors, remote sensing images have become widely used. In particular, images with both high spatial and spectral resolutions are highly desirable in various remote sensing applications, such as image classification, segmentation, object detection, etc. [1,2]. However, due to the technical limitations of the sensors and other imaging factors, such ideal images cannot be obtained directly [3]. Most Earth observation satellites, such as QuickBird, IKONOS, GeoEye-1, WorldView-2, etc., provide both high spatial resolution (HR) panchromatic (PAN) image with a low spectral resolution, and low spatial resolution (LR) multispectral (MS) image with a relative higher spectral resolution. The fusion process that makes full use of the complementary information from the PAN and MS images to produce HR MS image is referred to as pansharpening.

To date, a variety of pansharpening methods have been proposed. In general, most of the existing methods are based on a basic protocol, which can be summarized as: (1) determine the high spatial structure information, and it can be obtained from the PAN image by a tool such as a filter or other methods; and (2) inject the high spatial structure information into the MS image, based on a certain model. The image fusion methods based on this protocol can be sorted into several basic categories: arithmetic combination (AC)-based fusion methods, component substitution (CS)-based fusion methods, and multiresolution analysis (MRA)-based fusion methods. In addition, model-based fusion methods [4,5,6,7] have been developed in recent years; however, due to their complexity and time-consuming computations, these algorithms will not be discussed in detail in this paper.

Among the pansharpening methods described above, the AC-based fusion methods are the simplest. They are based on the arithmetic combination of the PAN and MS bands. The most representative are the Brovey fusion method [8] and the UNB-Pansharp fusion method [9], which has been successfully commercialized in the PCI Geomatica software. The CS-based algorithms are another popular pansharpening category; its basic idea is that the MS bands are firstly transformed into another new space with decorrelated components to reduce information redundancy, one of the components is then substituted by the HR PAN image to improve the spatial resolution of the MS image. The representative methods include the popular intensity-hue-saturation (IHS) fusion [10,11,12], the Gram-Schmidt (GS) fusion [13], principal component analysis (PCA) fusion [14], etc. In general, the AC-based fusion methods and the CS-based fusion methods can achieve the fused products with better spatial structures; however, they perform slightly poorer in the preservation of spectral information.

The MRA-based fusion methods are generally with relative less spectral distortions, though they are slightly sensitive to the spatial distortions. In general, they extract the high frequency information of the PAN image based on the wavelet transform [15,16,17,18] and the Laplacian pyramid [19,20], etc. In addition, the edge-preserving filters have been introduced into MRA-based image fusion algorithms [21,22,23,24]. In particular, the edge-preserving guided filter based fusion methods [25,26] have attracted an ever-increasing attention in recent years. To the best of our knowledge, Li et al. [25] were the first to introduce the guided filter into data fusion for multi-focus and multi-modal images, where the guided filter was used to construct the weight map between the layers of the source images. Joshi et al. [26] subsequently proposed an image fusion method using a multistage guided filter. However, most of the fusion algorithms using the edge-preserving filters decompose the PAN image into “low-frequency” (actually, the “low frequency” includes both the low-frequency and large-scale features) and detail information, without giving sufficient concern to the edge-preserving characteristics.

In this paper, a novel pansharpening method using a guided filter based on three-layer decomposition is proposed. The proposed algorithm is based on an MRA framework, and the PAN image is decomposed into a low-frequency layer, an edge layer, and a detail layer. The edge layer and the detail layer are then as the high spatial structures to be injected into the MS image by a proportional injection model. In addition, two new quantitative evaluation indices are developed.

The remainder of this paper is organized as follows. In Section 2, the guided filter is briefly reviewed. The proposed method is presented in Section 3. In Section 4, the experimental results and analyses are presented, and Section 5 concludes the paper.

## 2. Guided Filter

The guided filter is derived from a local linear model, it generates the filtering output by considering the content of a guidance image, and the guidance image can be either the input image itself or another different image. For convenience, we denote the guidance image as q, the input image as y, and the output image as O. The output image O is assumed to be a linear transformation of the guidance image q in a local window $\Omega_{k}$ centered at pixel k:

(1) $$O_{i}=a_{k}q_{i}+b_{k}\;\forall \;i\in \Omega_{k}$$

where $\left(a_{k},b_{k}\right)$ are linear coefficients, and i is the pixel location. It indicates that $\nabla O=a_{k}\nabla q$, which ensures that the output image O has an edge only when q has an edge. $a_{k}$ and $b_{k}$ can be solved by minimizing the difference between y and O:

(2) $$E(a_{k},b_{k})=\sum_{i\in \Omega_{k}}({\left(a_{k}q_{i}+b_{k}-y_{i}\right)}^{2}+\varepsilon {a_{k}}^{2})$$

here, ε is the regularization parameter to prevent $a_{k}$ from being too large.

For convenience, the guided filter can be also represented as:

(3) $$O_{i}=\sum_{j}w_{ij}(q)y_{j}$$

here, i and j are pixel indices, $w_{ij}$ is a kernel function of the guidance image q, and it is independent of the input image y. It is expressed as follows:

(4) $$w_{ij}=(1/|\Omega {|}^{2})\sum_{k:(i,j)\in \Omega_{k}}\left(1+(q_{i}-m_{k})(q_{j}-m_{k})/(\delta_{k}^{2}+\varepsilon )\right)$$

where $m_{k}$ and $\delta_{k}^{2}$ are the mean and variance of the guidance image in the window $\Omega_{k}$, respectively. After obtaining the kernel function, the output image O can be solved by Equation (3).

## 3. Proposed Method

### 3.1. Overview

**Figure 1:**
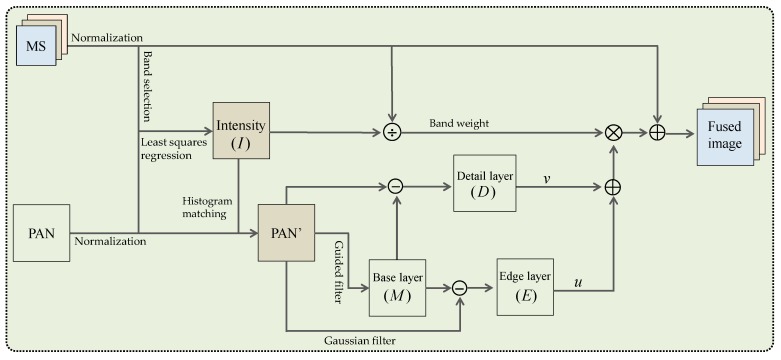
Schematic diagram of the proposed method.

The proposed pansharpening method is outlined in Figure 1. It is based on MRA framework by using the popular edge-preserving guided filter. In the proposed method, the PAN image is decomposed into three layers, i.e., the edge layer, the detail layer, and the low frequency layer, by considering the edge-preserving characteristics of the guided filter. The edge layer and detail layer are then injected into MS image by a proportional injection model [9,18,27,28,29]. The main processes of the proposed method are as follows:

(1) The pixel values of the original MS and PAN images are normalized to 0–1 to strengthen the correlation of the MS bands and PAN image. Then, histogram matching of the PAN image to the intensity component is performed, and the intensity component is a linear combination of the bicubic resampling MS, denoted as $\overset{⌢}{M}S$, whose spectral responses is approximately covered by the PAN [7,30]. Here, the linear combination coefficients are calculated by original MS and the downsampled PAN image with least square regression [31].
(2) The histogram-matched PAN image is decomposed into three layers, i.e., a strong edge layer E, a detail layer D, and a low-frequency layer L, based on three layer decomposition technique.
(3) The edge layer E and the detail layer D are injected into each MS band by a proportional injection model to obtain the fused image. It is represented as: $F_{b}=\tilde{M}S_{b}+W_{b}(u*E+v*D)$, where $F_{b}$ denotes the b-th band of the fused image, $\tilde{M}S_{b}$ is the anti-aliasing bicubic resampling MS image followed by guided filtering to suppress the spatial distortion, and here, the guidance image is the resampling MS image to preserve its original spectral information as much as possible. $W_{b}$ represents the b-band weight to determine the amount of high-frequency information to be injected, and it is represented as $W_{b}=\overset{⌢}{M}S_{b}/I$. The u and v are parameters to control the relative contribution of the edge layer and the detail layer, respectively.

### 3.2. Three-Layer Decomposition

**Figure 2:**
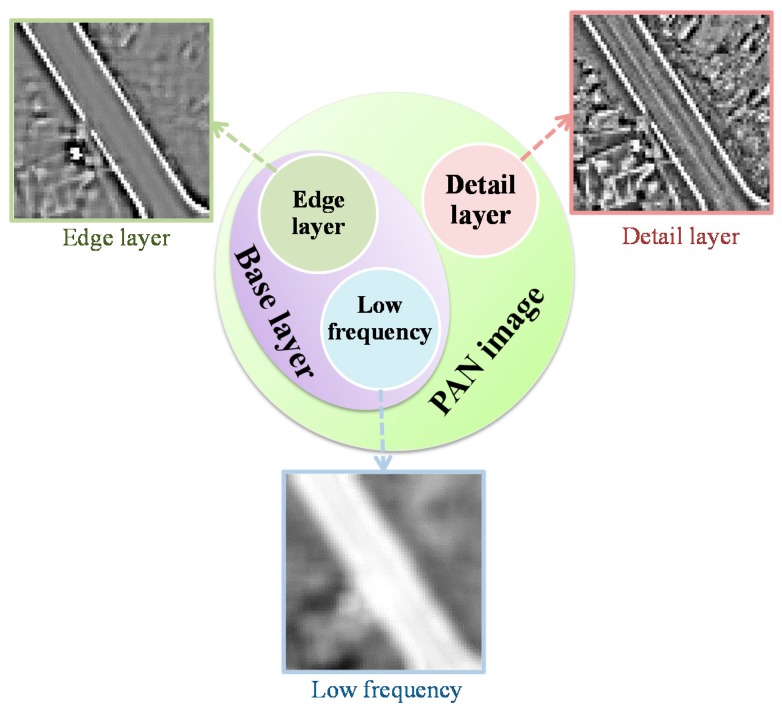
Schematic diagram of the three-layer decomposition.

The traditional pansharpening methods using edge-preserving filters generally decompose the PAN image into a “low-frequency” layer and a detail layer [21,22,23] by drawing from the way of traditional MRA-based fusion algorithms [15,16,17]; however, the decomposed “low-frequency” layer actually includes large-scale features. Bennett et al. [24] adopted a dual bilateral filter to fuse RGB and IR video streams, which decomposes the image into low frequencies, edges, and detail features. Inspired by this idea, a three-layer decomposition based on guided filter for pansharpening is proposed to split the PAN into a low-frequency layer, an edge layer, and a detail layer, as shown in Figure 2. The details are as follows:

(1) Firstly, the guided filter is applied to decompose the histogram-matched PAN image into a base layer and a detail layer.
  (5) $$M=G*P^{\prime }$$
  where M is the base layer, in which the low frequency layer and the strong edge layer are included. $P^{\prime }$ is the histogram-matched PAN image, and G denotes the guided filter. Here, the guidance image is consistent with the input image, i.e., the histogram-matched PAN. Once the base layer is obtained, the detail layer can be easily obtained by subtracting the base layer from the histogram-matched PAN image:
  (6) $$D=P^{\prime }-M$$
  where D denotes the detail layer.
(2) Then the strong edges are separated from the base layer, by reason that although the detail layer is obtained, there are still strong edges remaining in the base layer, which can be clearly seen in Figure 2. It is represented as:
  (7) $$E=M-g*P^{\prime }$$
  where E is the strong edge layer, g denotes the Gaussian low-pass filter, and the $g*P^{\prime }$ represents the low frequency layer of the PAN image.

## 4. Experimental Results and Analyses

In the experiments, several remote sensing satellite images including IKONOS, QuickBird, and GF-1 were utilized to comprehensively verify the effectiveness of the proposed method. In Wald’s [32] view, the synthetic image should be as similar as possible to the image that the corresponding sensor would observe at the highest spatial resolution; however, as there is no ideal reference images, the original PAN and MS images were firstly degraded to an inferior spatial resolution level by the ratio of the spatial resolution of the PAN and MS images, and then the original MS was treated as the reference image [7]. In addition, several state-of-the-art pansharpening methods were introduced for comparison, including Gram-Schmidt (GS) fusion method (implemented with ENVI 4.7, and GS1 was obtained by the average of the low-resolution MS files), principal component analysis (PCA) fusion method (implemented with ENVI 4.7), adaptive intensity-hue-saturation (AIHS) fusion method [33], and the additive wavelet luminance proportional (AWLP) method [18].

### 4.1. Quantitative Evaluation Indices

The proposed methods were verified from both qualitative and quantitative aspects. The qualitative evaluation involved analyzing the fused image directly from visual effects. To quantitatively analyze the fused image, several popular evaluation indices were used, i.e., the correlation coefficient (CC) [18,32], the spectral angle mapper (SAM) [34], the universal image quality index (UIQI) [35], the root-mean-square error (RMSE) [18], and the relative dimensionless global error in synthesis (ERGAS) [18,34,36]. In addition, two new quantitative evaluation indices, i.e., the modified correlation coefficient (MCC) and the modified universal image quality index (MUIQI), were developed in this paper, as shown in Table 1. Here, F denotes the fused image, R represents the reference image, and $\sigma_{V(F_{i,b=1...B})(R_{i,b=1...B})}$ denotes the covariance of the spectral bands in vector at the pixel position i. $N_{1}N_{2}$ represents the spatial dimension. In fact, the existing CC and UIQI are mainly focused on the evaluation of the radiance distortion; however, the developed MCC and MUIQI can be more comprehensively evaluated on both radiance distortion and interrelationship preservation among the spectral bands. In addition, to avoid the subjective evaluation from spectral profiles by selecting only few specific pixels in existing studies [7], the horizontal profiles of the column means for each band were introduced to more comprehensively and objectively evaluate the fused results.

### 4.2. Experimental Results

**Figure 3:**
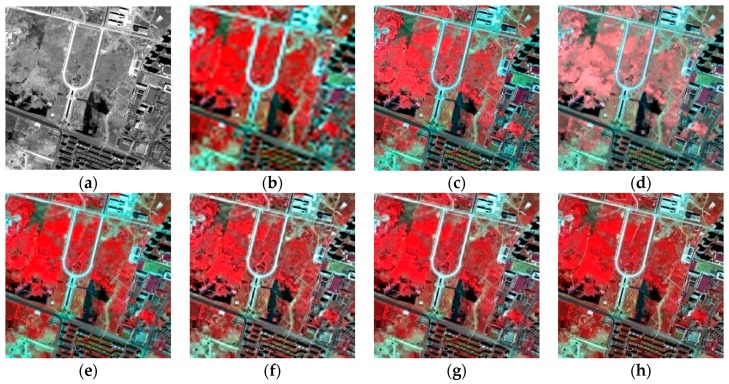
Fusion results of IKONOS experiment. (**a**) PAN image; (**b**) MS image; (**c**) GS fusion result; (**d**) PCA fusion result; (**e**) AIHS fusion result; (**f**) AWLP fusion result; (**g**) proposed fusion result; (**h**) original MS image.

**Figure 4:**
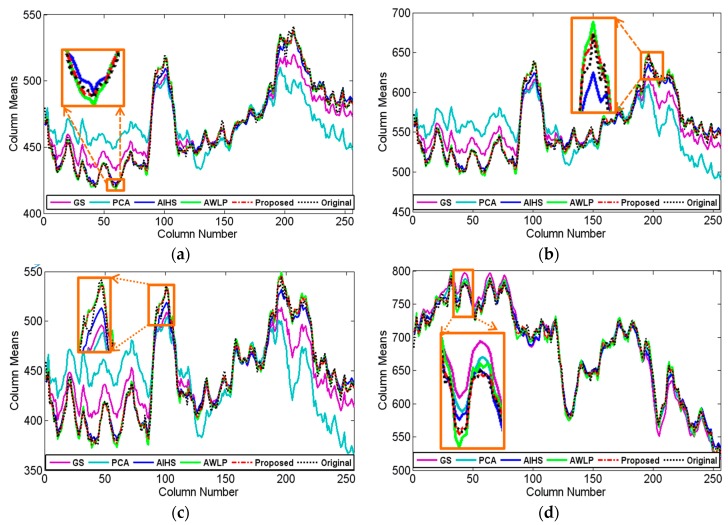
Horizontal profiles of the column means for the IKONOS fusion results. (**a**) band 1; (**b**) band 2; (**c**) band 3; (**d**) band 4.

The experiments were implemented on IKONOS, QuickBird, and GF-1 satellite images. Firstly, the IKONOS experiment is shown. Figure 3 shows the experimental results of the IKONOS satellite images from Huangshi City, Hubei Province, China. The proposed fusion result is shown in Figure 3g with the parameter u being 1.0 and v being 1.2, and the radius of the window size and the parameter ε of guided filter were empirically set to 2 and 0.01, respectively. Figure 3c,f show the fused results of the GS, PCA, AIHS, and the AWLP methods, respectively. It can be seen that the PCA fusion result generates obvious color distortion. In contrast, the spectral distortion of the GS fusion result is relatively smaller, indicating that the GS method is more stable than the PCA method for vegetation areas. Figure 4 shows that the profiles, especially bands 1–3, of GS and PCA fusion results are quite different to the profiles of the original image, which indicates the poor spectral information preservation of the two methods. For comparison, the AIHS and AWLP fusion results give relative better visual effects for spectral preservation; however, Figure 4 shows that some local sections of the AIHS and AWLP profiles have some degree of deviation from the original image. In contrast, the fusion result of the proposed method is the most similar to the reference image, and the spectral profiles of the proposed fusion result are also the closest to the reference image, which indicts the good spectral information preservation. Table 2 shows the quantitative evaluation results. This shows that only the CC and MCC values of the proposed method are 0.0001 and 0.0008 lower, respectively, than the best value; however, all the other indices of the proposed method are better than the other fusion methods. Therefore, it is demonstrated that the proposed method can obtain a higher spectral fidelity result with good spatial texture information.

**Figure 5:**
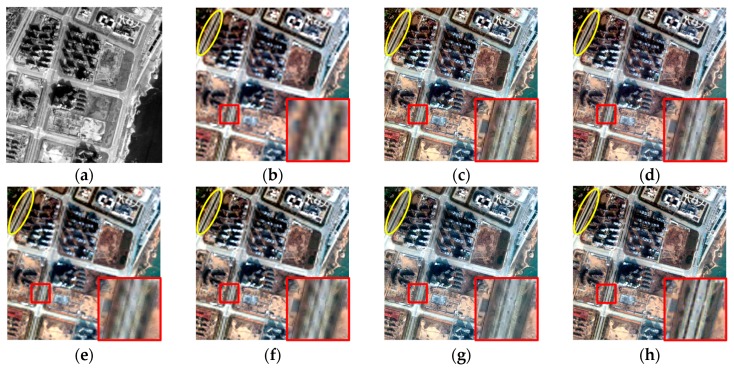
Fusion results of the QuickBird experiment. (**a**) PAN image; (**b**) MS image; (**c**) GS fusion result; (**d**) PCA fusion result; (**e**) AIHS fusion result; (**f**) AWLP fusion result; (**g**) proposed fusion result; (**h**) original MS image.

**Figure 6:**
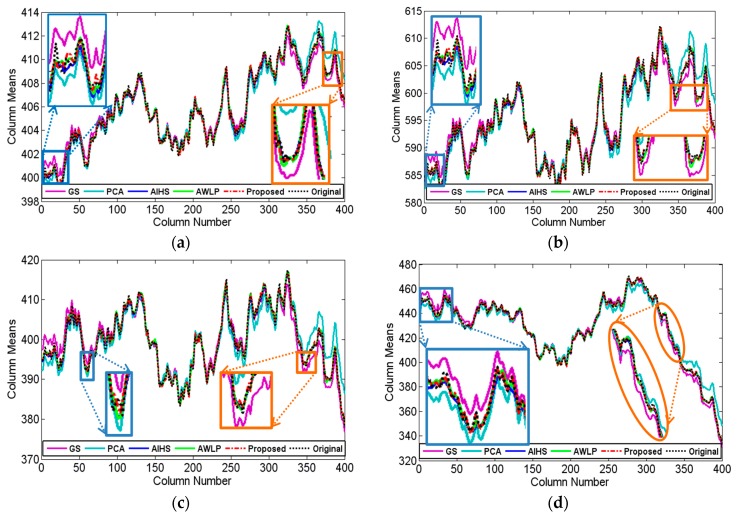
Horizontal profiles of the column means for the QuickBird fusion results. (**a**) band 1; (**b**) band 2; (**c**) band 3; (**d**) band 4.

The QuickBird experimental results are shown in Figure 5. The QuickBird PAN and MS images are located in Nanchang City, Jiangxi Province, China, and they were acquired on 8 October 2004. Figure 5g shows the proposed fusion result with the parameter u = 1.0 and v = 1.0. In addition, the radius of the window size and the parameter ε of guided filter were empirically set to 2 and 0.01, respectively. Figure 5c,f show the fused results of the GS, PCA, AIHS, and the AWLP methods, respectively. On the whole, all the methods can obtain good fused results. For comparison, the AIHS and the AWLP fusion results present slightly spatial distortions in this experiment. The proposed method can well suppress the spatial distortions, and it has better spatial visual effect and higher spectral fidelity. To evaluate the fusion result objectively, the horizontal profiles of the column means for each band are displayed in Figure 6. The black dotted line represents the original image, and the closer to the black dotted line, the better of the fused result. Figure 6 shows that there is a certain degree of deviation between the horizontal profiles of GS and PCA and the original image. The horizontal profiles of AIHS, AWLP, and the proposed method are closest to the original image, and the difference is small between them. To comprehensively compare the fusion methods, the quantitative indices are shown in Table 3. It shows that most of the evaluation indices for the proposed method are the best. The reason why some of the spectral indices from the PCA and GS methods are slightly better is that the two methods are relatively more stable for buildings and roads, which are the main features of the image. Overall, the proposed method, not only obtains a good spatial effect, but also has a higher spectral fidelity than other methods.

**Figure 7:**
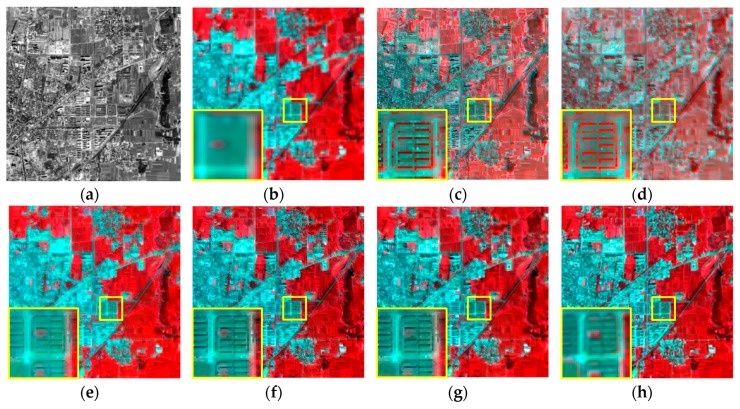
Fusion results of the GF-1 experiment. (**a**) PAN image; (**b**) MS image; (**c**) GS fusion result; (**d**) PCA fusion result; (**e**) AIHS fusion result; (**f**) AWLP fusion result; (**g**) proposed fusion result; (**h**) original MS image.

**Figure 8:**
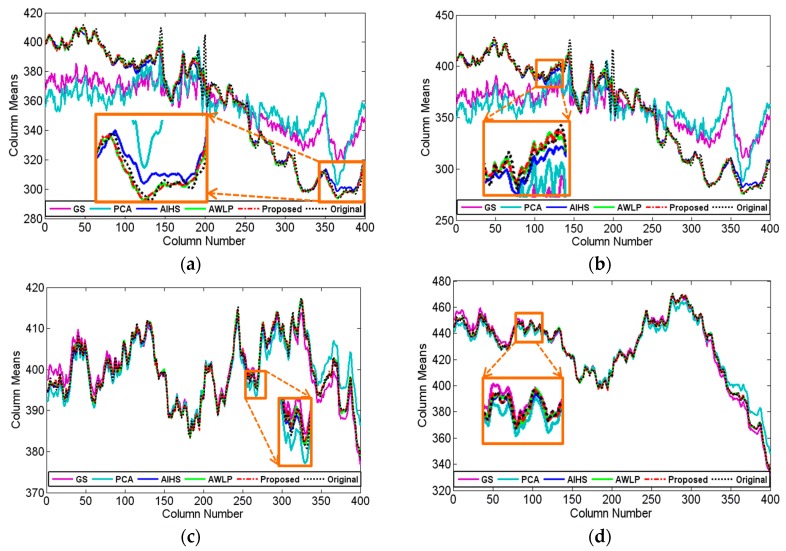
Horizontal profiles of the column means for the GF-1 fusion results by the different fusion methods. (**a**) band 1; (**b**) band 2; (**c**) band 3; (**d**) band 4.

Figure 7 shows the experimental results of GF-1 satellite images from Nanyang City, Henan Province, China, acquired on 6 August 2013. The parameter u was set to 1.0 and v was set to 0.9, the radius of the window size and the parameter ε of guided filter were empirically set to 2 and 0.01, respectively. It shows that the experimental results are similar with the IKONOS experiment. As with the IKONOS experiment in Figure 3c,d, the GS and PCA methods show serious spectral distortion in this GF-1 experiment. Visually, the color of AIHS, AWLP, and the proposed fusion result is the closest to the reference image. Figure 8 shows that the profiles of AWLP, AIHS, and the proposed fusion results are also the closest to the reference image, and it is hard to distinguish between them. Hence, to more objectively evaluate the fusion results, the quantitative indices of the fusion results are displayed in Table 4. It is shown that the proposed method has relative slight better fusion performance than other methods.

### 4.3. Discussion

This paper proposed a pansharpening method using an edge-preserving guided filter based on the three-layer decomposition, and it is different from the existing pansharpening method with the edge-preserving filters, which decomposes the PAN image into the “low frequency” layer (actually, the “low frequency” includes both the low-frequency information and large-scale features, as shown in Figure 2) and a detail layer. In this paper, the PAN image is decomposed into three layers by considering the edge-preserving characteristics.

**Figure 9:**
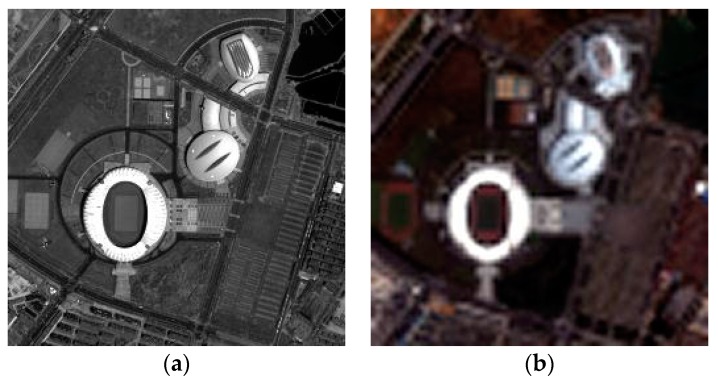
Experimental datasets in the validation of proposed pansharpening method based on three-layer decomposition over the traditional two-layer decomposition. (**a**) IKONOS PAN image; (**b**) IKONOS MS image with bicubic resampling.

**Figure 10:**
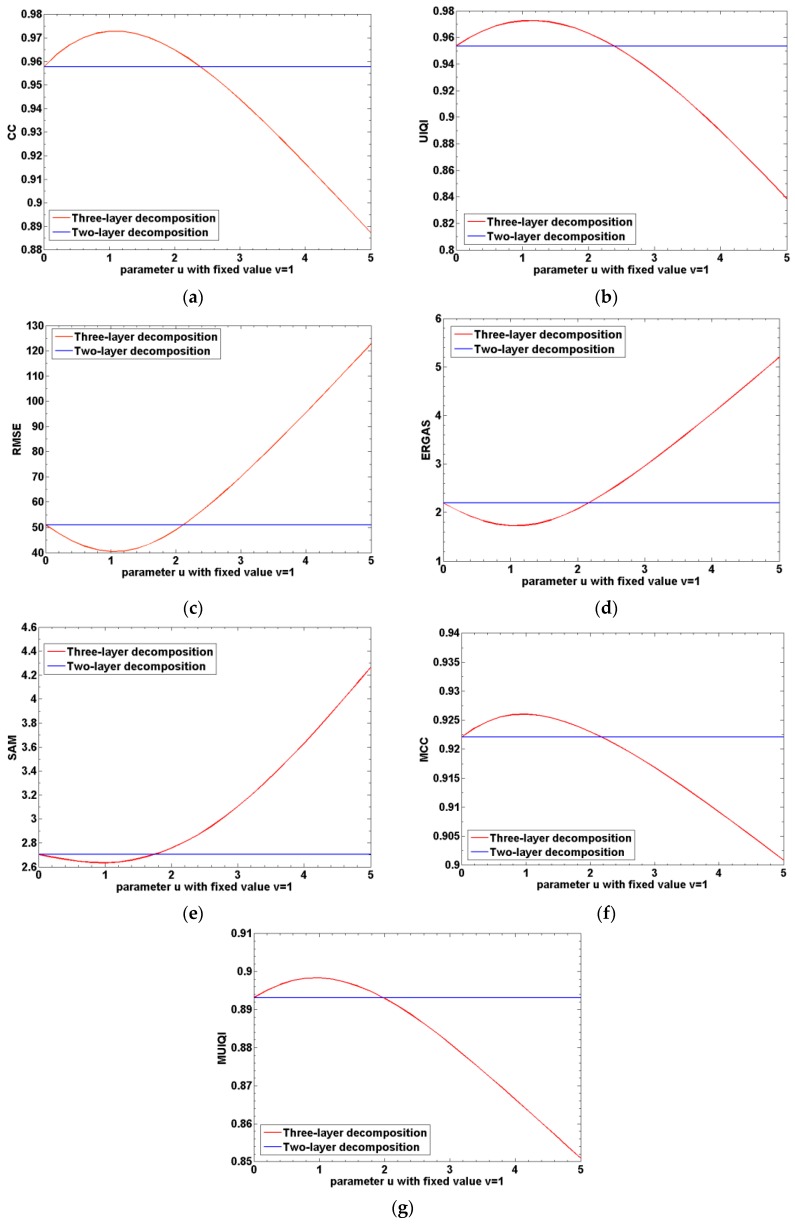
The statistical results for the comparison of the proposed three-layer decomposition to the two-layer decomposition. (**a**) Results of CC; (**b**) results of UIQI; (**c**) results of RMSE; (**d**) results of ERGAS; (**e**) results of SAM; (**f**) results of MCC; (**g**) results of MUIQI.

To verify the advantage of the proposed three-layer decomposition over the traditional two-layer decomposition, the statistical experimental results by using the three-layer and two-layer decomposition are shown. In this experiment, the IKONOS PAN (Figure 9a) and MS images (Figure 9b) are utilized, and statistical results of the CC, UIQI, RMSE, ERGAS, SAM, MCC, and MUIQI are shown in Figure 10. The blue curve denotes quantitative results of the traditional two-layer decomposition, and the red curve represents the statistical quantitative results by using the three-layer decomposition. Here, the abscissa denotes the different setting of parameter u with v being set to 1, indicating the different amount of injected edge layer. When the parameter u is 0, it denotes the result of two-layer decomposition.

It is shown that all the quantitative evaluation results can be improved with the increase of parameter u at first. This indicates that the proposed three-layer decomposition has better fusion results than the traditional two-layer decomposition as the injected edge layer within a certain degree. It is because that the traditional two-layer decomposition neglects the large-scale features, as clearly shown in Figure 2. On the whole, the three-layer decomposition has the advantage over the traditional two-layer decomposition.

## 5. Conclusions

This paper has presented a pansharpening method with an edge-preserving guided filter based on three-layer decomposition. In the proposed method, the PAN image is decomposed into three layers, i.e., the edge layer, the detail layer, and the low frequency layer, and then the edge layer and the detail layer are injected into the MS image by a proportional injection model. In addition, two new quantitative evaluation indices of MCC and MUIQI have been proposed. The proposed method is comprehensively verified by IKONOS, QuickBird, and GF-1 satellite images, and it is compared with several of the state-of-the-art pansharpening methods on both qualitative and quantitative aspects. The evaluation results confirm that the proposed three-layer decomposition for pansharpening, based on edge-preserving guided filter, is better than the traditional two-layer decomposition, and it can improve the spatial resolution while preserving the spectral fidelity.

## Figures and Tables

**Table 1:** Quantitative evaluation indices.

Evaluation Indices	Definitions	Meaning
CC [18,32]	$CC=\frac{1}{B}\sum_{b=1}^{B}\frac{\sigma_{F_{b},R_{b}}}{\sigma_{F_{b}}\sigma_{R_{b}}}$	the bigger the better
UIQI [35]	$UIQI=\frac{1}{B}\sum_{b=1}^{B}\frac{4\sigma_{F_{b}R_{b}}m_{F_{b}}m_{R_{b}}}{\left(\sigma_{F_{b}}^{2}+\sigma_{R_{b}}^{2})(m_{F_{b}}^{2}+m_{R_{b}}^{2}\right)}$	the bigger the better
RMSE [18]	$RMSE=\frac{1}{B}\sum_{b=1}^{B}\sqrt{\frac{||F_{b}-R_{b}|{|}_{F}^{2}}{N_{1}N_{2}}}$	the smaller the better
ERGAS [18,34,36]	$ERGAS=100\cdot \frac{h}{l}\cdot \sqrt{\frac{1}{B}\sum_{b=1}^{B}\frac{RMSE_{b}^{2}}{m_{R_{b}}^{2}}}$	the smaller the better
SAM [34]	$SAM=\frac{1}{N_{1}N_{2}}\sum_{i=1}^{N_{1}N_{2}}\cos^{-1}\frac{\sum_{b=1}^{B}\left(F_{i,b}\cdot R_{i,b}\right)}{\sqrt{\sum_{b=1}^{B}F_{i,b}^{2}\sum_{b=1}^{B}R_{i,b}^{2}}}$	the smaller the better
Proposed MCC	$MCC=\frac{1}{N_{1}N_{2}}\sum_{i=1}^{N_{1}N_{2}}\frac{\sigma_{V(F_{i,b=1...B}),(R_{i,b=1...B})}}{\sigma_{V(F_{i,b=1...B})}\sigma_{V(R_{i,b=1...B})}}$	the bigger the better
Proposed MUIQI	$MUIQI=\frac{1}{N_{1}N_{2}}\sum_{i=1}^{N_{1}N_{2}}\frac{4\sigma_{V(F_{i,b=1...B}),(R_{i,b=1...B})}m_{V(F_{i,b=1...B})}m_{V(R_{i,b=1...B})}}{\left(\sigma_{V(F_{i,b=1...B})}^{2}+\sigma_{V(R_{i,b=1...B})}^{2})(m_{V(F_{i,b=1...B})}^{2}+m_{V(R_{i,b=1...B})}^{2}\right)}$	the bigger the better

**Table 2:** Quantitative evaluation results of the IKONOS experiment (the best result is marked in bold, and the second best result is underlined).

Quality Indices	Ideal Value	Fusion Methods				
		GS	PCA	AIHS	AWLP	Proposed
CC	1	0.9370	0.8111	**0.9509**	0.9451	0.9508
RMSE	0	57.8762	86.2993	50.2334	53.6589	**47.6828**
UIQI	1	0.9129	0.7982	0.9381	0.9435	**0.9500**
ERGAS	0	2.7924	4.2949	2.4145	2.5517	**2.2823**
SAM	0	3.9072	6.0003	3.6110	3.5631	**3.4877**
MCC	1	0.9226	0.8546	0.9299	**0.9323**	0.9315
MUIQI	1	0.8869	0.8073	0.8958	0.8960	**0.8975**

**Table 3:** Quantitative evaluation results of the QuickBird experiment (the best result is marked in bold, and the second best result is underlined).

Quality Indices	Ideal Value	Fusion Methods				
		GS	PCA	AIHS	AWLP	Proposed
CC	1	0.9734	**0.9739**	0.9649	0.9691	0.9726
RMSE	0	9.4454	9.0217	10.5901	9.4798	**8.5592**
UIQI	1	0.9665	0.9715	0.9609	0.9688	**0.9723**
ERGAS	0	0.5842	0.5649	0.6608	0.5811	**0.5163**
SAM	0	0.7240	0.7766	0.7524	0.7004	**0.6851**
MCC	1	0.9964	0.9962	0.9960	**0.9965**	0.9962
MUIQI	1	**0.9958**	0.9956	0.9954	0.9954	**0.9958**

**Table 4:** Quantitative evaluation results of the GF-1 experiment (the best result is marked in bold, and the second-best result is underlined).

Quality Indices	Ideal Value	Fusion Methods				
		GS	PCA	AIHS	AWLP	Proposed
CC	1	0.6072	0.4019	0.9308	0.9226	**0.9326**
RMSE	0	63.756	80.3908	28.3677	29.731	**27.7250**
UIQI	1	0.5959	0.3974	0.9258	0.9221	**0.9317**
ERGAS	0	4.9706	6.0991	2.1309	2.1918	**2.0526**
SAM	0	6.9835	9.4628	2.4487	2.4777	**2.4206**
MCC	1	0.7970	0.7454	0.9336	0.9347	**0.9349**
MUIQI	1	0.7159	0.6342	0.9036	0.9042	**0.9054**
